# Molecular epidemiology and phylogenetic insights of lumpy skin disease in cattle from diverse agro-ecological regions of Punjab, Pakistan

**DOI:** 10.1371/journal.pone.0315532

**Published:** 2025-01-13

**Authors:** Muhammad Haider Jabbar, Farhan Ahmad Atif, Muhammad Kashif, Ishtiaq Ahmed, Fabrizio Iarussi, Ayman A. Swelum

**Affiliations:** 1 Department of Clinical Sciences, Medicine Section, College of Veterinary and Animal Sciences, Jhang, Pakistan; 2 Sub-Campus of University of Veterinary and Animal Sciences, Lahore, Pakistan; 3 Department of Pathobiology, Pathology Section, College of Veterinary and Animal Sciences, Jhang, Pakistan; 4 Department of Precision and Regenerative Medicine and Jonian Area (DiMePRe-J), Section of Veterinary Science and Animal Production, University of Bari Aldo Moro, Valenzano, Bari, Italy; 5 Department of Animal Production, College of Food and Agriculture Sciences, King Saud University, Riyadh, Saudi Arabia; Benha University Faculty of Veterinary Medicine, EGYPT

## Abstract

Lumpy skin disease (LSD) is an emerging, highly contagious transboundary disease of bovines caused by the Lumpy skin disease virus (LSDV), responsible for substantial economic losses to the dairy, meat, and leather industries in Pakistan as well as various countries around the world. Epidemiological information on LSD is scarce in Punjab, Pakistan. Therefore, a molecular epidemiological study was conducted in two agro-ecologically diverse districts (Bhakkar and Jhang) of Punjab, Pakistan. A total of 800 blood samples were randomly collected from the jugular vein of clinically suspected cattle with nodular lesions using a multistage cluster sampling technique. The sampling unit was indigenous, crossbred, and exotic breeds of cattle. Four hundred samples were collected from each district. Ten union councils (UC) were selected from each district, and two villages were selected from each union council. From each village, twenty cattle were selected for sample collection. The PCR-based overall prevalence of LSDV in clinically suspected cattle using the *P32* gene was 36.25% (36.25%; 290/800). The multivariable logistic regression analysis indicated that animals who were not treated with acaricide (*P* = 0.014; OR = 1.459; C.I = 1.079–1.972), body condition score (emaciated animals; *P* = 0.019; OR = 1.573; CI = 1.076–2.301), and gender (female; (*P =* 0.016; OR = 1.435; CI = 1.072–1.969) were significantly at higher risk for LSDV infection in cattle. The phylogenetic insights revealed that our isolates were linked to Kenya, China, Russia, Egypt, India, Zimbabwe, Iraq, and Iran. It can be concluded that LSD is widely distributed in the study area, with evidence of genetic diversity. Further studies are required on genetic composition using variable genetic markers for effective control and eradication of LSDV in Pakistan.

## Introduction

In Pakistan, the livestock industry is an imperative source of earnings for 8 million rural families, deriving 35–40% of income. Livestock farming contributed 14.36% to the national Gross Domestic Product (GDP) and 62.68% in agriculture during the fiscal year 2023. There are about 55.5 and 45.0 million cattle and buffaloes in Pakistan, respectively [1]. Undoubtedly, this sector faces multiple issues in the form of transboundary diseases such as lumpy skin disease.

The lumpy skin disease is an acute viral, highly contagious, emerging, vector-borne transboundary infection caused by the lumpy skin disease virus (LSDV), a DNA virus that belongs to the *Capripox* virus of the *Poxviridae* family [2]. LSDV mainly affects cattle and buffaloes [3], but may also spread to other wild ruminants, including giraffes, impalas, springboks, and Arabian Oryx [4]. *Capripox* virus strains cause sheep and goat pox. In 1929, LSD was first noticed in Zambia, and it then moved to South Africa and North Sudan [5]. The LSD was first detected in Pakistan in 2022 and rapidly spread, affecting a large population of bovines [6]. Lesions caused by nodules and lumps on the skin eventually result in decreased milk output, impaired meat quality, and affect animal hides [7,8]. In Southeast Asia and South Asia, the LSD has caused a loss of 1.45 billion dollars. The morbidity rate of LSD ranges from 3–85% [9], while the mortality rate is often less than 10% [10]. According to the survey of Sindh’s Livestock and Fisheries Department conducted in March 2022, it revealed that LSD resulted in a decrease in milk and meat by 60–70% [7]. The disease has also been gaining traction in Europe and Asia since 2012, and several epidemics have occurred across Europe [11]. In Asia, the first infection was noticed in Lebanon in 2012, later reported in Iraq, then in Jordan, Bangladesh, China, India, and Sri Lanka [4].

Summer time and humid weather are connected with a higher incidence of illness, which is correlated with an abundance of insect vectors [12]. The incubation period is 8 to 13 days after transmission between suspected and infected animals [5]. Outbreaks coincide with the vector season, especially in summer, which is favorable for the growth and multiplication of vector ticks. Vector transmission and non-vector transmission are the two forms of transmission that might happen. Direct or indirect non-vector transmission is both possible. Different body secretions, such as oral, nasal, ocular, milk, and semen, can directly transmit disease. Infected ticks and needles cause indirect transmission [12]. Fly, lice, and flea vectors that come from infected animals and automobiles can spread the infection [13]. After a brief period of viremia, the LSD virus replicates inside the skin and mucous membranes. Resulting in skinillness, weight loss, decreased milk yields, abortion, poor reproductive function, damage to the hide, and loss of draught power [11]. Vasculitis, necrotic epidermis, and eosinophilic intra-cytoplasmic inclusion bodies are the hallmarks of LSD infection in terms of clinical signs [14]. Skin nodules ranging in size from 10 to 55 mm in diameter and an increased body temperature (> 104.9°F) are typical symptoms of LSD [13]. Edema appears on the neck, brisket, and lower legs, which causes lameness [5]. The primary effects of LSD are on daily milk output, hides, and meat quality.

Knowledge on the epidemiology and genetic structure of LSDV (*P32 gene*) is not fully investigated in Punjab, Pakistan. Earlier, limited published research differed in terms of sample size and study area. Various genes have been targeted for the molecular characterization of LSDV, such as RP030, LSDV142, IL10LP, GPCR, and P32 genes [15–17]. The molecular characterization is important from an epizootiological and disease control standpoint. Therefore, the current study was aimed at determining the molecular prevalence, risk factors, and phylogenetic investigations of lumpy skin disease in blood collected from LSD-suspected cattle from agro-ecologically diverse regions of Jhang and Bhakkar districts of Punjab, Pakistan.

## Materials and methods

### Ethics statement

The study was approved by the Ethical Review Committee of the College of Veterinary and Animal Sciences, Jhang, vide letter No. CVAS/ERC-110, dated July 17, 2023.

### Study area

**Fig 1:**
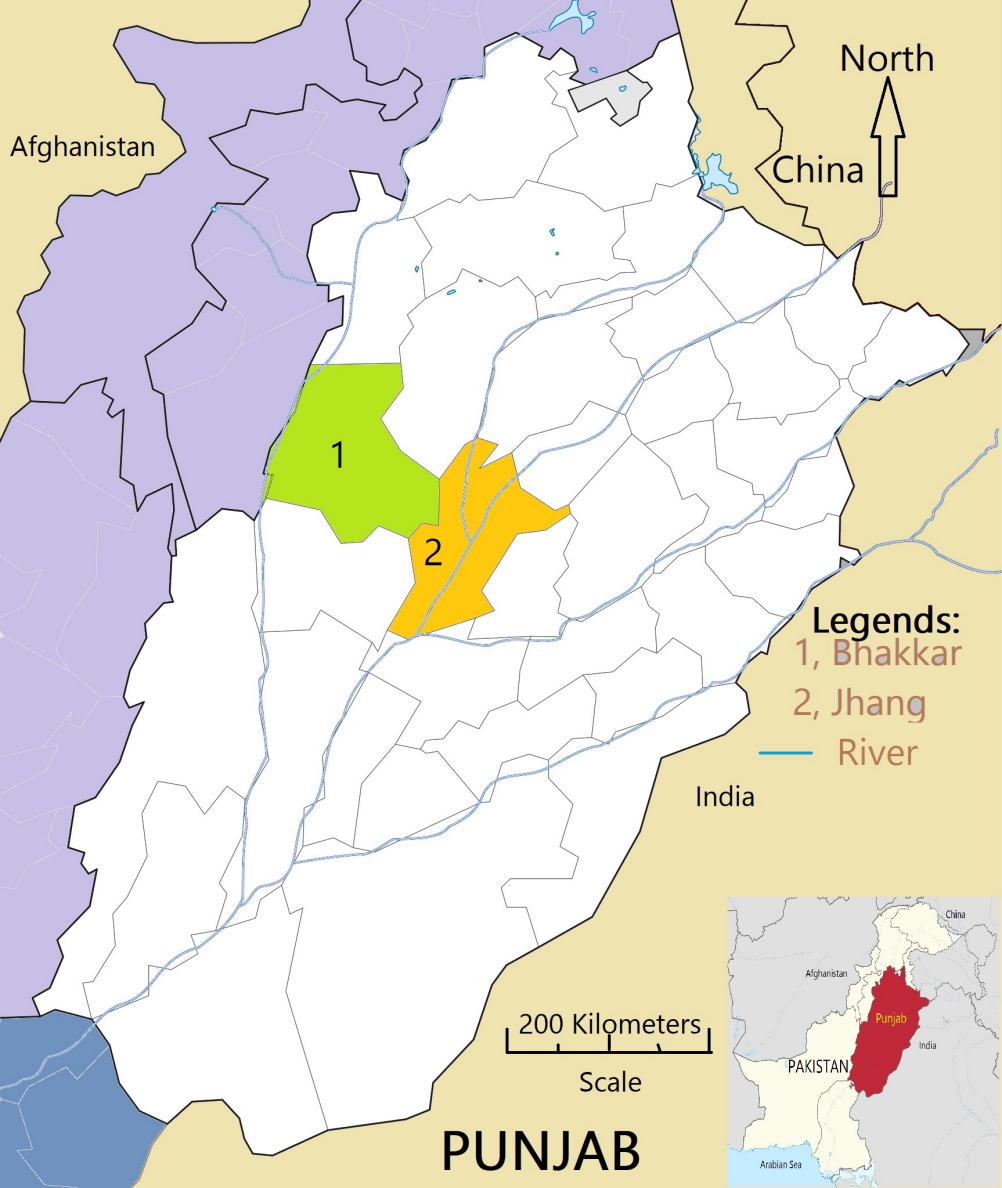
Map of Jhang and Bhakkar districts of Punjab, Pakistan.

The Jhang and Bhakkar districts were selected based on different agro-climatic environments (Fig 1). Major agro-climatic conditions related to these regions are topography, solar radiation, vegetation cover, temperature, rainfall, and humidity. The Jhang district is located at 31.27 latitude, 72.33 longitude, and it is situated at an elevation of 158 meters above sea level. The annual rainfall ranges from 7–10 inches (180 mm). Whereas, the district of Bhakkar is situated between the Indus River and Chenab River, coordinated at 31°37’60N 71°4’0E at an elevation of 159 meters above sea level, and lies on the left bank of the Indus River. Moreover, the annual amount of precipitation in Bhakkar is 213 mm.

### Study animals and samples

A total of 800 blood samples were randomly collected from suspected cattle with nodular lesions from the jugular vein belonging to different breeds of cattle (exotic, indigenous, and cross-bred) using a multistage cluster sampling technique. A sample size of 384 was calculated, assuming 50% prevalence in each district using the formula as mentioned by Thrusfield [18]. For convenience, four hundred samples were collected from each district. Ten union councils were selected from each district, and two villages were selected from each union council. From each village, twenty cattle were selected for sample collection. Aseptically, 3 ml of blood was collected from each animal and placed in EDTA vacutainers. The samples were transported on a cold chain avoiding direct sunlight. The samples were transferred to the Post-Graduate Laboratory of Medicine, College of Veterinary and Animal Sciences, Jhang, Pakistan, for further analysis.

### DNA extraction and PCR amplification of the LSDV p32 gene

The DNA extraction was performed using the Thermo Scientific GeneJet Genomic DNA Purification Kit according to the manufacturer’s guidelines [19]. The LSDV was detected using PCR, utilizing primers as described by [20]. The pair of primers P32-F (5′-TCGTTGGTCGCGAAATTTCAG-3′) and P32-R (5′-GAGCCATCCATTTTCCAACTCT-3′) targeted a 759-bp fragment. A total of 25μl of mixtures were used to prepare the PCR reactions, containing 10 nmol of forward primer (1 μl) as well as reverse primers (1 μl), including 5 μl of DNA, 12.5 μl of master mix (Deam Taq PCR Master Mix, Thermo Scientific™, USA; Catalog no. K1081), and 5.5 μl of nuclease-free water. Using the thermal cycler (BIO RAD T100^TM^), amplification was carried out after a 5-minute denaturing period at 95°C, followed by 35 cycles of incubation at 95°C for 45 seconds, 56°C for 45 seconds, and 72°C for 65 seconds. The final extension was performed at 72°C for 5 minutes. A 1.5% agarose gel was utilized for electrophoresis of the PCR product, and the gel was envisaged with a UV illuminator.

### Risk factors estimation

For assessment of epidemiological risk factors, a pretested questionnaire was filled out to gather data after consent at the time of sample collection from each animal. The data concerning the biotic (host and pathogenic) and abiotic (area, housing, and managemental) including geographic region (Jhang and Bhakkar), gender (male and female), age (<1 year, 1–3 years, and >3–6 years), breed (crossbred, local, and exotic), grazing system (separate and communal), insect vectors (present and absent), farm type (dairy and beef), acaricide use (yes and no), and body condition score (obese, moderate, and emaciated) associated with LSD were collected. The closed-ended questionnaire was filled out on the spot at the time of blood sampling from each animal (S1 File).

### Sequencing and phylogenetic analysis of LSDV

The representative positive samples (n = 4) were sent for nucleotide sequencing to Celemics Inc. (Seoul, South Korea) using Barcode-tagged sequencing (BTSeqTM). Two sequences were selected from each region. The current sequences of LSDV isolated from cattle were utilized for the construction of a phylogenetic tree and compared with isolates from different countries, such as **Saudi Arabia** MN422449, MN422450; **Kenya** MW452625, MW452626; **Egypt** MN18201, MN18202, MN271753, MN271754, MN271755; **Russia** MH029289, MH029291; **Iran** KX960769, KX960770, KX960771, KX960778; **Iraq** KR066462, KR066463, KR066464; **Nepal** OL689595, OL689596, OL689598, OL689599; **Australia** AF124516; **China** MN598005, OL977074, OM046584; **Zimbabwe** KX033504, KX033506; **India** MT074104, MT074105; **South Africa** MT552113, MT552114; **Bangladesh** PP277532, PP277533 and PP277534; **Pakistan** OP807845, OP807846, OP807847, OP807848, and OP807849.

The maximum likelihood approach and the Tamura 3-parameter model were used to infer the evolutionary history [21]. The most likely tree was presented. The initial tree(s) for the heuristic search were generated automatically by applying the Neighbor-Join and BioNJ algorithms to a matrix of pairwise distances computed using the Tamura 3 parameter model. This study included 44 different nucleotide sequences. The phylogenetic tree was constructed in MEGA11 [22]. Statistical support for the inside braces was set for bootstrap analysis with 1000 replications.

### Statistical analysis

A chi-square test was performed to determine the association of LSD prevalence with disease determinants. Univariable analysis was used to analyze the relationship between the occurrence of LSD in cattle and variables of geographic regions (Bhakkar and Jhang), gender, age, breed, grazing system, insect vectors, farm type, acaricide use, and body condition score. Multivariable analysis was performed for the selected variables, which had a *p*-value < 0.2, to determine odds ratios and confidence intervals for each significant variable. A *p*-value less than or equal to 0.05 was considered significant. The statistical data was analyzed with IBM SPSS Statistics 26.

## Results

**Fig 2:**
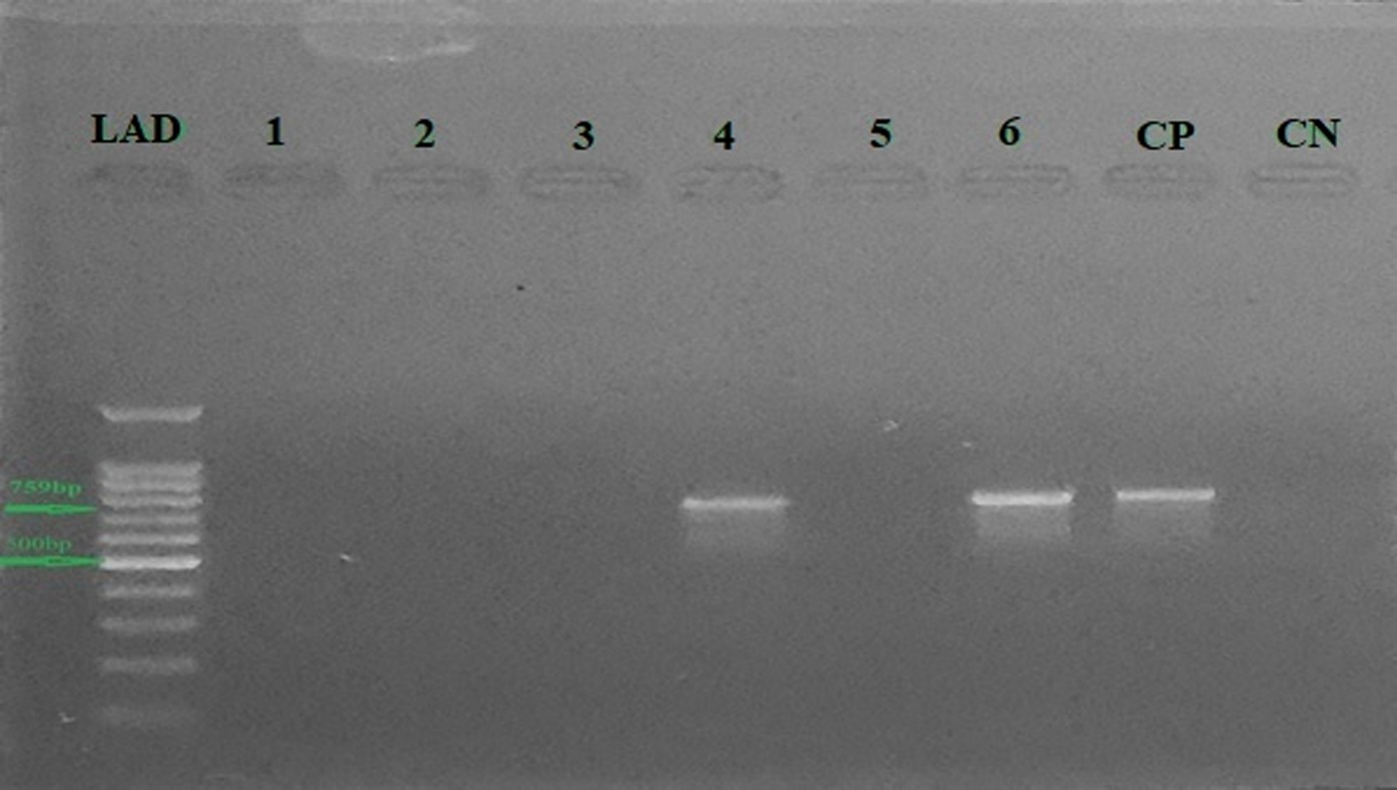
The PCR-based gel image of lumpy skin disease using the P32 gene. Wells 4 and 6 are showing positive results. Whereas well 7 was considered as control positive (CP), and well 8 acted as control negative (CN).

The overall PCR-based prevalence of LSD in cattle from the Bhakkar (38.75%; 155/400) and Jhang (33.75%; 135/400) districts was 36.25% (290/800) (Table 1). A non-significant association (*P* > 0.05) was observed between geographic regions. The length of 759 bp was observed using a 100 bp ladder (LAD) as shown in Fig 2. Major clinical signs observed in the study animals were fever, nasal discharge, ocular discharge, salivation, enlarged pre-scapular and pre-femoral lymph nodes, and skin nodules on the head, neck, trunk, perineum, udder, and teats. The lowest prevalence was recorded in exotic breeds (22.72%) compared to crossbred (48.00%) and local breed (31.02%) cattle with a significant association (*P* = 0.000, *X*^*2*^ = 31.233, df = 2). The data was transformed and assessed for collinearity. The variables of the presence of vectors and farm type were removed after diagnosis of collinearity. A total of 321 cattle were treated with acaricide regularly and 479 cattle never received any acaricide. The highest prevalence was observed in the group without any use of acaricidal drug with a significant statistical association (*P* = 0.021, *X*^*2*^ = 5.314, df = 2). It was found that females were more infected with LSDV (39.34%) compared to males (31.41%). The role of gender proved to be a significant association (*P* = 0.023, *X*^*2*^ = 5.184, df = 1). According to the grazing system, this variable indicated a significant association with LSDV infection (*P* = 0.032, *X*^*2*^ = 4.595, df = 1). The animals that were kept intensively had lower infection rates (32.46%) than those kept in the communal grazing system (39.75%). On the basis of body condition score, animals were divided into three categories (obese, moderate, and emaciated). Sick animals expressed higher levels of infection compared to healthy animals. Disease infectivity was highest in emaciated animals (41.72%) compared to moderate (34.20%) and obese animals (31.53%), and this determinant expressed a significant association (*P* = 0.043, *X*^*2*^ = 6.277, df = 2). Nevertheless, chi-square analysis revealed that prevalence based on age had a non-significant association. The occurrence of the disease was lower in younger (< 1 year, 32.54%) than in those of the intermediate (> 1–3 years, 36.79%) and adults (> 3–6 years, 37.50%), respectively (Table 2).

**Table 1:** Prevalence of LSD in cattle from Jhang and Bhakkar districts of Punjab, Pakistan.

District	No. of cattle examined	No. of LSD-affected cattle	Prevalence (%)
Bhakkar	400	155	38.75
Jhang	400	135	33.75
Overall	800	290	36.25

**Table 2:** Chi-square analysis of risk factors associated with molecular prevalence of LSD in cattle from Jhang and Bhakkar districts of Punjab, Pakistan.

Variables	Categories	Total	Positive	Prevalence (%)	Chi-square (*X*^*2*^)	*p*-value	df
Geographic regions	Jhang	400	135	33.75	2.164	0.141	1
	Bhakkar	400	155	38.75			
Gender	Male	312	98	31.41	5.184	0.023*	1
	Female	488	192	39.34			
Age	<1 year	169	55	32.54	1.305	0.521	2
	1–3 years	231	85	36.79			
	>3–6 years	400	150	37.50			
Breed	Crossbred	300	144	48.00	31.233	0.000*	2
	Local	390	121	31.02			
	Exotic	110	25	22.72			

*Statistically significant; df (degree of freedom).

### Risk factors analysis

Univariable analysis revealed that gender (*P =* 0.020, OR = 1.452, CI = 1.062–1.985), no use of acaricide (*P =* 0.027, OR = 1.421, CI = 1.041–1.938), and body condition score (*P* = 0.021, OR = 1.255, CI = 1.034–1.524) as significant risk factors with strong statistical evidence. Whereas, non-significant or inverse association was noticed for variables such as geographic area, gender, age breed and grazing system (Table 3). All determinants with a *p*-value of <0.2 in the univariable analysis were incorporated into the multivariable model. Nonetheless, multivariate regression analysis indicated that animals who were not treated with acaricide (*P* = 0.014; OR = 1.459; CI = 1.079–1.972), body condition score of cattle (emaciated animals; *P* = 0.019; OR = 1.573; CI = 1.076–2.301), and gender (female; (*P =* 0.016; OR = 1.435; CI = 1.072–1.969) were significantly at higher risk for LSDV infection in cattle (Table 4).

**Table 3:** Univariate analysis of risk factors associated with molecular prevalence of LSD in cattle from Jhang and Bhakkar districts of Punjab, Pakistan.

Variable	B^1^	*p*-value	Odds ratio	95% Confidence Interval (CI)	
				Lower CI	Lower CI
Geographic region	0.022	0.892	1.022	.747	1.399
Gender	0.373	0.020	1.452	1.062	1.985
Age	0.104	0.293	1.110	.914	1.347
Breed	-0.607	0.000	0.545	.431	.688
Grazing system	-0.220	0.153	0.802	.593	1.086
Acaricide use	0.351	0.027	0.027	1.421	1.041
Body condition score	0.227	0.021	0.021	1.255	1.034

^1^Regression coefficient; CI (Confidence interval)

*Statistically significant.

**Table 4:** Multivariate analysis of risk factors associated with molecular prevalence of LSD in cattle from Jhang and Bhakkar districts of Punjab, Pakistan.

Variable (Category)	B^1^	*p*-value	Odds ratio	95% Confidence Interval (CI)	
				Lower CI	Upper CI
Acaricide^2^ use (No use)	0.378	0.014*	1.459	1.079	1.972
Body condition score (Obese)	—	0.034	—	—	—
Body condition score (Moderate)	0.104	0.595	1.109	.757	1.626
Body condition score (Emaciated)	0.453	0.019*	1.573	1.076	2.301
Gender^3^ (Female)	0.374	0.016*	1.453	1.072	1.969

B^1^Regression coefficient; CI (Confidence interval)

*Statistically significant; Reference categories:

^2^Acaricide use (yes), and

^3^gender (male).

### Sequencing and phylogenetic analysis of LSDV

**Fig 3:**
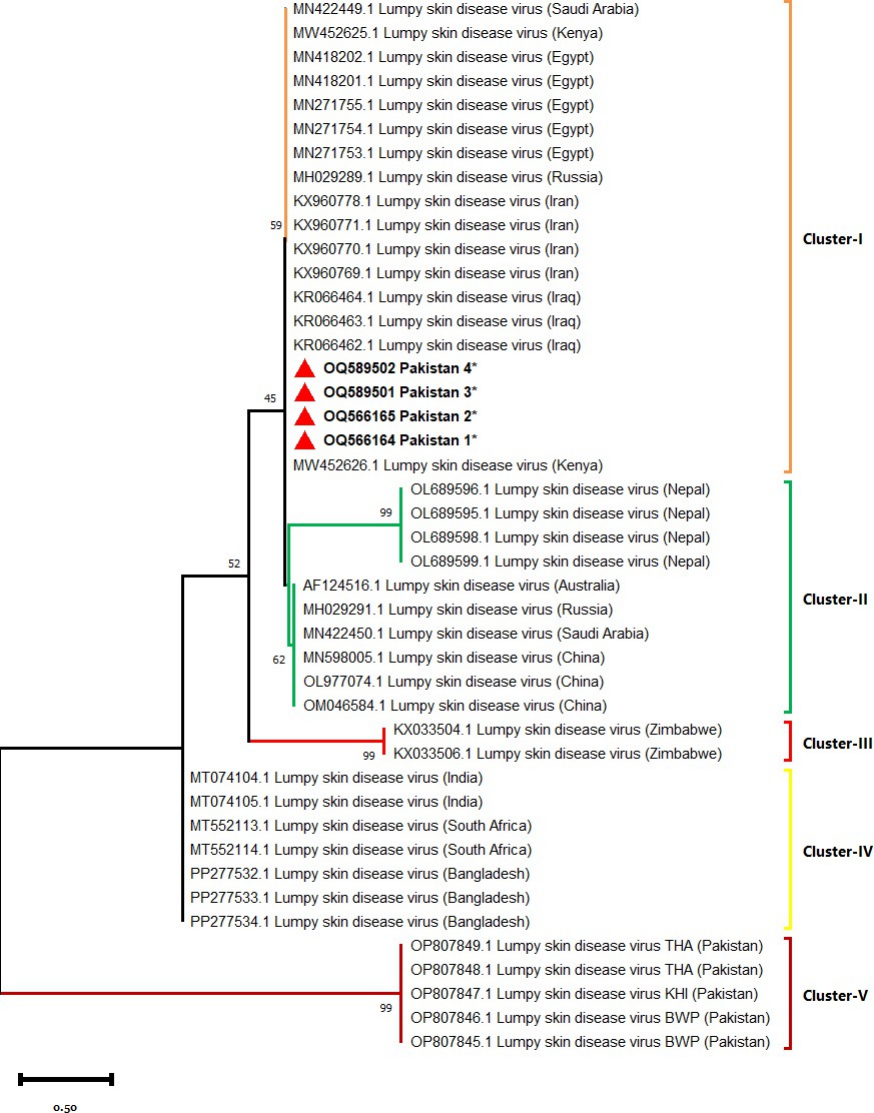
Maximum likelihood based phylogenetic tree of LSDV collected from cattle using the P32 gene.

We successfully gained GenBank accession numbers for four isolates of LSDV isolated from cattle (OQ566164, OQ566165, OQ589501, and OQ589502). The maximum likelihood method-based phylogenetic tree of LSDV (*P32 gene*) revealed five major clusters. Cluster-I contained isolates of the current study and isolates from **Saudi Arabia:** MN422450; **Kenya:** MW452625, MW452626; **Egypt:** MN18201, MN18202, MN271753, MN271754, MN271755; **Russia:** MH029289, MH029291; **Iran:** KX960769, KX960770, KX960771, KX960778 and **Iraq:** KR066462, KR066463, KR066464. Cluster-II consisted of isolates from **Nepal:** OL689595, OL689596, OL689598, OL689599; **Australia:** AF124516; **Russia:** MH029291; **Saudi Arabia:** MN422450 and **China:** MN598005, OL977074, OM046584. Cluster-III comprised of isolates from Zimbabwe KX033504 and KX033506. The isolates of **India** MT074104, MT074105; **South Africa** MT552113, MT552114; **Bangladesh** PP277532, PP277533, PP277534 were in cluster-IV. Whereas, the isolates collected from Southern Pakistani regions of Thatta, Karachi, and Bahawalpur were in Cluster-V. The isolates belonging to Iraq (KR066462, KR066462), Iran (KX960769, KX960770, KX960771, and KX960778), Egypt (MN18201, MN18202, MN271753, MN271754, and MN271755); Saudi Arabia and Nepal (OL689595, OL689596, OL689598, OL689599) were closer to current isolates followed by South African, Indian, and Bangladeshi isolates. However, the earlier Pakistani isolates were divergent from current isolates (Fig 3). Our sequence newly obtained in this study is highlighted in bold with a red triangle and asterisk. Our sequences based on the *P32* gene from cattle showed 99–100% resemblance with universal isolates deposited to GenBank. The phylogenetic analysis revealed that our isolates are novel and linked to Kenya, China, Russia, Egypt, India, Zimbabwe, Iraq and Iran.

## Discussion

LSD is a highly significant and economically important transboundary viral disease. Knowledge on the epidemiology and genetic structure of LSDV is lacking in diverse agro-climatic regions of Punjab, Pakistan. The current study focused on molecular detection, estimation of risk factors, and phylogenetic investigation of LSDV using *P32* in cattle from different agro-ecological regions. The P32 gene has undergone extensive conservation within capripoxviruses. This gene can yield sequence information that can be used to distinguish goat pox virus (GTPV), sheep pox virus (SPPV), and LSD viruses. This gene has been utilized as a diagnostic tool for LSDV, SPPV, and GTPV by various researchers [23–26].

The overall prevalence of LSD was 36.25%. Our findings are in close alignment with the study of Sudhakar and colleagues [4]. An Indian study based on RT-PCR reported a prevalence of 37.66%. Nevertheless, Abera and colleagues depicted a lower infectivity of 6.43% in Ethiopia by using an immunofluorescence antibody test with 99% specificity [27]. The occurrence of disease was higher in Bhakkar than in Jhang. However, the infection rate was statistically non-significant.

This study revealed a higher prevalence in crossbred cattle that were at higher risk of exposure due to extensive housing and poor-quality feeding. Poor hygienic housing conditions also contributed to the higher incidence of LSDV. Our study coincides with the previously reported prevalence as mentioned by Hasib and coworkers [28], showing a higher prevalence in cross-bred cattle. Our findings are in line with the study of Selim and co-scientists [29], who reported that crossbred cattle were significantly at higher disease risk. The prevalence of crossbred cattle was 48.00% compared to local (31.02%) and exotic breeds (22.72%). Crossbred cattle might be more susceptible owing to lesser disease resistance to ectoparasites than native breeds [30]. Our findings are partially in line with the research of Kiplagat and colleagues [27], who reported exotic breeds with higher occurrence compared to hybrid ones.

The risk factor study based on age revealed that LSD was higher in adult cattle (3–6 years) compared to young (<1 year) and intermediate groups (1–3 years). The current infection rates in the study are quite well supported by Abera and collaborators [31], where the disease positivity rates were 8.78%, 5%, and 2.74% in adults, young, and calves, respectively. Similarly, Sameea and coworkers [20] also depicted the highest morbidity rate in adults (73.3%) compared to cattle of less than 6 months of age (39.3%). This might be due to the fact that the adults share the same pasture and water sources. Young calves might stay at the owner’s home, thus having less exposure to ticks and other vectors [31]. Conversely, our study is not in agreement with Sameea et al. [20], who mentioned that disease frequency rates in young and adults were 26% and 17%, respectively. This might be due to malnourished and immune-compromised young ones.

The prevalence on the basis of farm type was not found to be statistically different, but the disease rate was recorded to be higher in dairy animals (38.43%) compared to beef cattle (32.67%). This might be due to high ambient temperatures and farm management designed to provide a higher milk yield, which may cause stress and exacerbate the condition [30]. Our study was in agreement with Selim and collaborators [29], who depicted that dairy animals show a higher prevalence (18.3%) compared to beef breeds (17.2%).

In the present work, acaricide treatment of cattle showed a higher association with LSD. Those animals receiving regular acaricide treatment were affected at a lesser frequency (30.15%) than those who were regularly treated with acaricide (31.46%), while animals that were never treated with acaricide showed a higher prevalence (39.45%). This might be due to the lesser density of vector (flies, ticks, and mosquitoes) infestation in animals than in those who were subjected to acaricide use. The vectors play a role in the mechanical transmission of the virus, as endorsed by Teshome and Derso [32]. Nonetheless, this determinant was least frequently discussed by other researchers.

In the current study, chi-square analysis based on gender expressed a statistically non-significant association. In male, the infection rate was 31.42%, while in females the morbidity rate was 39.34%. Our findings are in agreement with Khalil and coworkers [33], who mentioned that the prevalence in females was 11% and in males the occurrence of the disease was 5%. Our results are coherent with those of Selim and collaborators [29], where in females, the disease rate was 18.8%, and in the males, the infection rate was 15%. Kasem and contributors stated that the higher incidence of LSD in female cattle may be related to their exposure to a variety of stressful situations, such as pregnancy, deprivation of nutrition, and parturition [34]. Our research is contrary to Gammada et al. [35], who stated that the positivity of the disease is higher in males than females due to stress of exhaustion or fatigue.

There was a statistically substantial association between the intensive and communal grazing systems. In the communal grazing system, the prevalence was 39.75% and in the separate feeding system, the prevalence was 32.46%. The findings of our study are aligned with those of Selim et al. [29], where the prevalence was higher in communal grazing (20.9%) than in intensive grazing (14.6%). Our present research also validates the findings of Hailu et al. [36], where it showed a higher occurrence in communal grazing compared to separate grazing. In communal grazing, a total of 376 herds were tested; 171 herds were infected with LSD, while in separate grazing, 2 herds were positive out of 17 herds.

Current univariate regression analysis indicated that gender, no use of an acaricidal application, and body condition score were identified as significant risk factors. Whereas multivariate regression analysis indicated that animals who were not treated with acaricides, the body condition score of cattle (emaciated animals) and gender (female) were significantly at higher risk for LSDV infection in cattle. Whereas the Thailand-based study indicated that herds kept for more than five years and the absence of insect control management were important disease determinants [37]. Nonetheless, Selim and associates revealed Holstein breed, adult cattle, summer season, introduction of new animals, communal grazing, communal watering points, and cattle in contact with other animals are the important risk factors in Northern Egypt [29]. Risk factors vary from region to region. Multiple risk factors, such as environmental (area, climate, temperature, rainfall [36], humid agro-climate, high density of vector population, habitat, rainy season/humidity, altitude, vegetation cover); host (sex, breed, age, presence of vectors, health status) and managemental factors (flock size, introduction of new animals, housing, floor, communal grazing system, drinking stations/points, animal movement, animal contact during milking, contaminated fomites) are associated with the occurrence of lumpy skin disease [29,33–40].

The phylogenetic understandings revealed that our isolates were linked to Iraq, Iran, Russia, Egypt, Kenya, Saudi Arabia, and Nepal. Whereas, divergent from India, South Africa, Bangladesh, and Pakistan. Further studies need to be conducted on genetic composition using variable genetic markers. A large-scale survey is required for the assessment of the epidemiological status and economic impact of LSD for effective monitoring and control of LSDV in Pakistan.

## Conclusions

The phylogenetic analysis revealed that the isolates in the current study are linked to Kenya, China, Russia, Egypt, India, Zimbabwe, Iraq, and Iran. The LSDV is widespread in the study area, with evidence of genetic diversity. The emaciated female cattle that were not treated with acaricides are at greater risk for LSDV infection. Further studies need to be conducted on genetic composition using variable genetic markers for effective prevention and control of LSDV in Pakistan.

## Supporting information

S1 File Questionnaire. (DOCX)
