# Supplementary material for: Molecular epidemiology and phylogenetic insights of lumpy skin disease in cattle from diverse agro-ecological regions of Punjab, Pakistan
Source: PLoS One. 2025 Jan 13;20(1):e0315532. doi: 10.1371/journal.pone.0315532 (PMC11729989; doi:10.1371/journal.pone.0315532)
Supplement: S1 File — (DOCX) [file pone.0315532.s001.docx]

**QUESTIONNAIRE**

**Study tittle:**

**Molecular epidemiology and phylogenetic analysis of lumpy skin disease virus in cattle from Jhang and Bhakkar districts.**

**Muhammad Haider Jabbar**

**Scholar M. Phil in Clinical Medicine**

**BIOTIC FACTORS (Living)**

**Sample type:** Blood

**Age:** < 1 year, >1 to ≤3 year or >3 to 6 year___________

**Gender:** Male, female_____________________________

**Breed:** Local, cross or exotic________________________

**Insect vectors:** Present and absent ___________________

**Health status:** Good, moderate or poor _______________

**Farm type:** Dairy or beef __________________________

**ABIOTIC FACTORS (Non-living)**

**Area:** Jhang or Bhakkar district_____________________

**Acaricide use:** Yes or No__________________________

**Grazing system:** Separate or communal_______________
